# A Privacy-Preserving Multi-Task Learning Framework for Face Detection, Landmark Localization, Pose Estimation, and Gender Recognition

**DOI:** 10.3389/fnbot.2019.00112

**Published:** 2020-01-14

**Authors:** Chen Zhang, Xiongwei Hu, Yu Xie, Maoguo Gong, Bin Yu

**Affiliations:** ^1^School of Computer Science and Technology, Xidian University, Xi'an, China; ^2^Key Laboratory of Intelligent Perception and Image Understanding of Ministry of Education, School of Electronic Engineering, Xidian University, Xi'an, China

**Keywords:** multi-task learning, privacy preserving, differential private stochastic gradient descent, balance different learning tasks, differential privacy guarantees

## Abstract

Recently, multi-task learning (MTL) has been extensively studied for various face processing tasks, including face detection, landmark localization, pose estimation, and gender recognition. This approach endeavors to train a better model by exploiting the synergy among the related tasks. However, the raw face dataset used for training often contains sensitive and private information, which can be maliciously recovered by carefully analyzing the model and outputs. To address this problem, we propose a novel privacy-preserving multi-task learning approach that utilizes the differential private stochastic gradient descent algorithm to optimize the end-to-end multi-task model and weighs the loss functions of multiple tasks to improve learning efficiency and prediction accuracy. Specifically, calibrated noise is added to the gradient of loss functions to preserve the privacy of the training data during model training. Furthermore, we exploit the homoscedastic uncertainty to balance different learning tasks. The experiments demonstrate that the proposed approach yields differential privacy guarantees without decreasing the accuracy of HyperFace under a desirable privacy budget.

## 1. Introduction

Recently, neurorobotics has made great progress in a wide range of scientific fields, including locomotion and motor control, learning and memory systems, action selection and value systems, and many more. All of these models need to consider the problem of simultaneously solving multiple related tasks, which is the prevalent idea behind multi-task learning (MTL). MTL focuses on learning several tasks simultaneously by transferring knowledge among these tasks. In training machine learning models, the required datasets may contain private and sensitive information. Privacy is considered the private sphere of an individual or group that secludes information about themselves from the public environment and ought to be preserved adequately. These datasets for machine learning tasks enable faster commercial or scientific progress, but privacy-preservation has become an urgent issue that needs to be addressed. In early works, some privacy-preserving techniques, including k-anonymity (Sweeney, 2002), l-diversity (Machanavajjhala et al., 2006), and t-closeness (Li et al., 2007), that anonymize the data before analyzing it, were proposed. Even though curators can apply several simple anonymization techniques, sensitive personal information still has a high probability of being disclosed (Wang et al., 2010). As an essential and robust privacy model, differential privacy can successfully resist most privacy attacks and provide a provable privacy guarantee (Dwork, 2011a; McMahan et al., 2017; Wang et al., 2018; Erlingsson et al., 2019). Moreover, differentially private MTL was introduced by Gupta et al. (2016), where the authors proposed a differentially private algorithm using a noisy task relation matrix and developed an attribute-wise noise addition scheme that significantly improves the utility of their proposed method. However, those algorithms significantly increase the time complexity of MTL, making it difficult to perform the iterative calculation in training models.

MTL is widely used in a broad range of practical applications, including face detection (Ranjan et al., 2017; Ahn et al., 2018; Chen et al., 2018; Zhao et al., 2019), federated MTL (Smith et al., 2017; Corinzia and Buhmann, 2019; Sattler et al., 2019), speech recognition (Huang et al., 2013; Kim et al., 2017; Liu et al., 2017; Subramanian et al., 2018), and other applications (Doersch and Zisserman, 2017; Han et al., 2017; Liu et al., 2017, 2019; Hessel et al., 2019). Ranjan et al. (2017) presented an algorithm for simultaneous face detection, landmark localization, pose estimation, and gender recognition. The proposed method, called HyperFace, exploits the synergy among the tasks to boost their individual performance. Their work demonstrates that HyperFace is able to capture both global and local information regarding faces and performs significantly better than many competitive algorithms for each of these four tasks. However, multi-task models without privacy preservation may impair the privacy of users during the training process of models. Therefore, enforcing privacy preservation on private datasets is a challenge that needs to be addressed. Existing privacy preservation methods have successfully integrated differential privacy into iterative training processes like stochastic gradient descent (Abadi et al., 2016; Papernot et al., 2016; McMahan et al., 2017; Wu et al., 2017; Bun et al., 2018; Wang et al., 2018). These differentially private frameworks preserve private and sensitive data within an acceptable performance range in single-task models. However, up until now, there have been few studies on privacy preservation in MTL. Another major challenge is that a reasonable trade-off of multi-task losses can make the noise level more balanced among individual tasks. Previous methods (Sermanet et al., 2013; Eigen and Fergus, 2015; Kokkinos, 2017) always manually adjust weights or just initialize weights and often become trapped in a local optimum.

As mentioned above, MTL has made great progress in a wide range of practical applications. However, an important challenge is how to preserve private and sensitive information contained in training datasets. In practice, existing privacy preservation methods have been successfully applied to many single-task models, but they are rarely applied to multi-task models. In this paper, we integrate the rigorous differential privacy mechanism with a multi-task framework named HyperFace through training five related tasks within a desirable privacy budget. We adopt the differential private stochastic gradient descent algorithm to optimize the end-to-end multi-task model. Specifically, Gaussian noise is added to the gradient of loss functions for preserving the privacy of the training data during the training process of the model. Furthermore, we exploit the homoscedastic uncertainty to weigh loss functions of multiple tasks, which can improve learning efficiency and prediction accuracy. Our main contributions are summarized as follows:

1. We propose a novel privacy-preserving multi-task learning framework that provides differential privacy guarantees on HyperFace.
2. The loss functions of multiple tasks are adjusted by utilizing the homoscedastic uncertainty, which makes the model more balanced within the privacy budget on individual tasks.
3. We evaluate our approach on face detection, landmark localization, pose estimation, and gender recognition. The extensive experiments demonstrate that data privacy can be preserved without decreasing accuracy.

The rest of the paper is organized as follows. The next section reviews differential privacy and multi-task learning. Section 3 describes the proposed approach in detail. Section 4 analyzes the experimental results of our approach, and section 5 concludes the paper.

## 2. Related Work

In this section, we briefly review differential privacy and multi-task learning.

### 2.1. Differential Privacy

Differential privacy is a new and promising model presented by Dwork et al. (2006b) in 2006. It provides strong privacy guarantees by requiring the indistinguishability of whether or not an individual's data exists in a dataset (McSherry and Talwar, 2007; Dwork, 2011b; Dwork and Roth, 2014; McMahan et al., 2017; Wang et al., 2018; Erlingsson et al., 2019). We regard a dataset as *d* or *d*′ on the basis of whether the individual is present or not. A differential privacy mechanism provides indistinguishability guarantees with respect to the pair (*d, d*′); the datasets *d* and *d*′ are referred to as adjacent datasets. The definition of (ε, δ)-differential privacy is provided as follow:

** DEFINITION 1**. *A randomized mechanism* M:D→R *satisfies (ε, δ)-differential privacy if, for any two adjacent datasets* $d,d^{\prime }\in D$ *and for any subset of outputs* Y⊆R, *it holds that*

$$Pr\left[M\left(X\right)\in Y\right]\leq e^{\varepsilon }Pr\left[M\left(X^{\prime }\right)\in Y\right]+\delta$$

The parameter ε denotes the privacy budget, which controls the privacy level of M. For a small ε, the probability distributions of the output results of M on *d* and *d*′ are extremely similar, and it is difficult for attackers to distinguish the two datasets. In addition, the parameter δ, which provides a possibility to violate ε-differential privacy, does not exist in the original definition of ε-differential privacy (Dwork et al., 2006a).

There are several common noise perturbation mechanisms for differential privacy that mask the original datasets or intermediate results during the training process of models: the Laplace mechanism, the exponential mechanism, and the Gaussian mechanism. Phan et al. (2017) developed a novel mechanism that injects Laplace noise into the computation of Layer-Wise Relevance Propagation (LRP) to preserve differential privacy in deep learning. Chaudhuri et al. (2011, 2013) adopted the exponential mechanism as a privacy-preserving tuning method by training classifiers with different parameters on disjoint subsets of the data and then randomizing the selection of which classifier to release. In Yin and Liu (2017), numerical evaluations of the Gaussian cumulative density function are used to obtain the optimal variance to improve the utility of output perturbation Gaussian mechanisms for differential privacy.

To add less noise, the gradient computation of loss functions samples Gaussian noise instead of Laplacian noise, since the tail of the Gaussian distribution diminishes far more rapidly than that of the Laplacian distribution. A general paradigm for approximating the deterministic real-valued function f:M→ℝ with a differential privacy mechanism is via additive noise calibrated to *f*'s sensitivity *S*_*f*_, which is defined as the maximum of the absolute distance |*f*(*d*) − *f*(*d*′)| where *d* and *d*′ are adjacent datasets. For instance, the Gaussian noise mechanism is defined by

$$M\left(d\right)≜f\left(d\right)+N\left(0,S_{f}^{2}\cdot \sigma^{2}\right)$$

where $N\left(0,S_{f}^{2}\cdot \sigma^{2}\right)$ is the normal (Gaussian) distribution with mean 0 and standard deviation *S*_*f*_σ.

### 2.2. Multi-Task Learning

MTL is an interesting and promising area in machine learning that aims to improve the performance of multiple related learning tasks by transferring useful information among them. Based on an assumption that all of the tasks, or at least a subset of them, are related, jointly learning multiple tasks is empirically and theoretically found to lead to better performance than learning them independently. Recently, MTL is becoming increasingly popular in many applications, such as recommendation, natural language processing, and face detection. Yin and Liu (2017) proposed a pose-directed multi-task convolutional neural network (CNN), and most importantly, an energy-based weight analysis method to explore how CNN-based multi-task learning works. However, multi-task learning algorithms may cause the leakage of information from different models across different tasks. Specifically, an attacker can participate in the multi-task learning process through one task, thereby acquiring model information of another task. To address this problem, Liu et al. (2018) developed a provable privacy-preserving MTL protocol that incorporates a homomorphic encryption technique to achieve the best security guarantee. Xie et al. (2017) proposed a novel privacy-preserving distributed multi-task learning framework for asynchronous updates and privacy preservation. Previous methods always apply privacy preservation to the parameters of models. In this paper, we combine HyperFace with a differential privacy mechanism for preserving the privacy of original datasets.

## 3. Methodology

This section presents our approach of differentially private learning on HyperFace, which provides a (ε, δ)-differential privacy guarantee for HyperFace. Section 3.1 summarizes the definition of the problem that needs to be resolved and the notations used, section 3.2 introduces the details of the framework, while section 3.3 discusses and analyzes the method.

### 3.1. Review of the Problem and Notations

HyperFace is a prevalent multi-task model for simultaneously learning the related tasks of face detection, landmark localization, pose estimation, and gender recognition. In this model, the synergy between related tasks is utilized to improve the performance of the individual tasks. There are two main problems for preserving privacy and boosting model performance in Hyperface. In practice, facial datasets used to train Hyperface contain a large amount of private and sensitive information. Training data without a strong privacy guarantee can be maliciously recovered by carefully analyzing the model and outputs. Another problem is that the performance of a multi-task model is highly dependent on appropriate weights among the loss of each task. However, HyperFace simply initializes these weights, which may cause the model to become trapped in a local optimum rather than reaching the global optimum. The notations and symbols used throughout the paper are summarized in Table 1.

**Table 1:** Notations and symbols.

**Notations**	**Descriptions**
(ε, δ)	Privacy budget
L(·)	General loss function with parameters
$g_{t}\left(x_{i}\right),{\bar{g}}_{t}\left(x_{i}\right)$	Gradient and bounded gradient of the *i*^*th*^ example in a subset of examples *L*_*t*_
*ĝ*_*t*_	Noisy gradient of a subset of examples
||·||_2_, *S*	ℓ_2_ norm of the gradient of an example
N(·)	Normal Gaussian distribution
η_*t*_	Learning rate of a subset of examples
loss_*_	Corresponding loss functions of different tasks

### 3.2. Our Approach

In this paper, we present a novel approach called Differentially Private Learning on HyperFace (DPLH) to preserve the privacy of original facial datasets that contain landmark coordinates, pose estimations, gender information, and much more. To collect the faces with private attributes that need to be protected, we need to crop all faces from each given image in facial datasets. When optimizing the loss function of each task with the stochastic gradient descent algorithm, we allocate a reasonable privacy budget across each of the gradient updates on examples and analyze the privacy cost of the trained model. To trade off the privacy and utility of the Hyperface multi-task model, we utilize the synergy between related tasks to adjust the weights of each loss function.

#### 3.2.1. Pre-training

There are two pre-training steps that need to be performed before the model update on Hyperface by applying the Gaussian mechanism: regional candidate selection and initializing the weights of HyperFace.

Facial datasets usually involve a large amount of private information that is potentially distributed over the images. In order to apply the differential privacy mechanism to these facial data, the given images are selectively cropped to generate positive candidate regions with faces and negative candidate regions without faces by regional candidate selection. We filter out candidate regions as positive and negative by computing the Intersection over Union (IOU) overlap. The candidate regions are considered as positive with an IOU overlap of more than 0.5, and negative candidate regions have an IOU overlap of <0.35. Subsequently, these selected candidate regions are scaled to 227 * 227 pixels as the input of the model. In addition, the ground truths, such as landmark localization and the visibility factor corresponding to these candidate regions, need to be adjusted as well since they are relative to the original images rather than the selected regions.

Initializing the weights of network is helpful for finding global optimal solutions or avoiding becoming trapped in poor local optimal solutions. A good initialization facilitates gradient propagation in deep networks and avoids the problems of a vanishing gradient or gradient exploding. In this paper, we pre-train a single-task model, whose parameters are initialized to the default, for face detection with an input of the candidate regions generated by regional selection. Then, the parameters of this single task are used to initialize HyperFace for better convergence performance.

#### 3.2.2. Training

Training data may not be effectively protected by only adding noise to the final parameters that result from the training process. Generally, there are few useful and exact characterizations of the dependence of these parameters on the training data. Moreover, adding excessive noise to the parameters may destroy the utility of the learning model. In the worst case, excessive noise will degrade the model performance, and a small amount of noise may not provide a strong privacy guarantee. Hence, we propose a novel approach for HyperFace to preserve the privacy of training data and control the influence of training data in the stochastic gradient descent computation.

**Figure 1:**
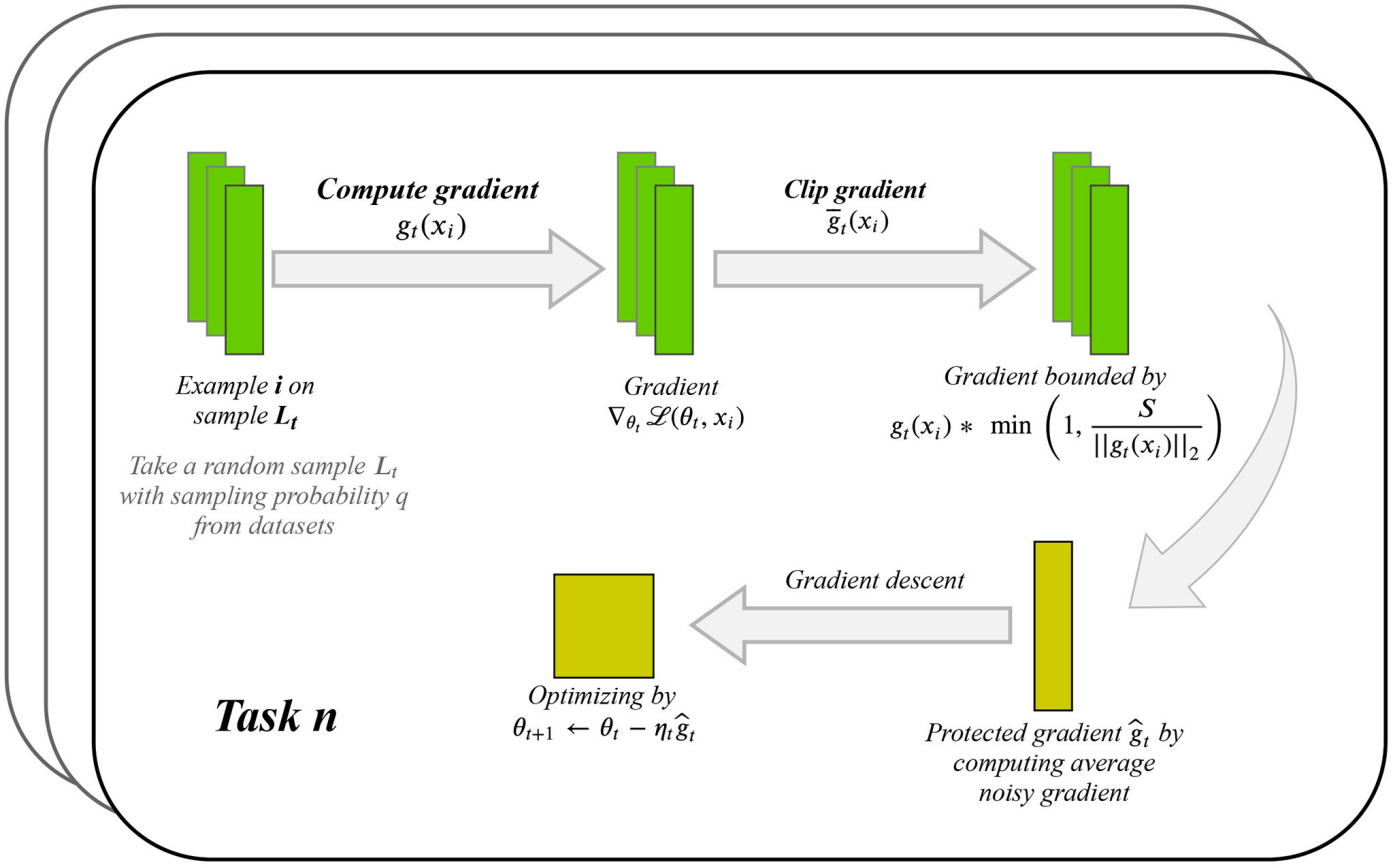
Per iterative computation process for preserving privacy on each learning task.

In the training process of our DPLH model, we iteratively compute the gradient update from training data and then apply the Gaussian mechanism for differential privacy to the gradient update. Figure 1 shows the per iterative computation process for protecting privacy while learning each task. Suppose the training datasets with *N* examples consist of selected candidate regions with adjusted ground truth. Given a sampling probability *q*, clipping threshold *S*, and noise multiplier *z*, our approach focuses on minimizing each task loss function $L\left(\theta^{j}\right)$ with parameter θ^*j*^(1 ≤ *j* ≤ 5) in the training process by using a stochastic gradient descent optimizing algorithm. At each step of stochastic gradient descent, we select a subset of the examples *L*_*t*_ ⊆ [1, …, *N*] by choosing each example with probability *q*. We compute the gradient $\nabla_{\theta^{j}}L\left(\theta^{j},x_{i}\right)$ as *g*_*t*_(*x*_*i*_) with each example *i* ∈ *L*_*t*_, clip each gradient to have maximum ℓ_2_ norm *S* using ${\bar{g}}_{t}\left(x_{i}\right)=g_{t}\left(x_{i}\right)*min\left(1,\frac{S}{||g_{t}\left(x_{i}\right)|{|}_{2}}\right)$, then add noise to them and compute the average of the noisy gradients by ${ĝ}_{t}=\frac{1}{qN}\left(\underset{i}{\sum }{\bar{g}}_{t}\left(x_{i}\right)+N\left(0,\sigma^{2}I\right)\right)$. Subsequently, we take a step in the opposite direction of this average noisy gradient, like θ_*t*+1_ = θ_*t*_ − *η*_*t*_*ĝ*_*t*_. In addition to outputting the model, we estimate the privacy budget of an iterative Gaussian noise mechanism by privacy accounting. We describe our approach in more detail below.

**Loss functions**. In order to better measure the performance of the model, different loss functions and evaluation metrics are used for the training tasks of face detection, landmark localization, landmark visibility, pose estimation, and gender classification.

*Face detection*. We use regional candidate selection to generate positive candidate regions with faces (*l* = 1) and negative candidate regions without faces (*l* = 0) in given images. We can train the face detection task with loss function *loss*_*D*_, given as follows

(1) $$\begin{array}{l} loss_{D}=\left(1-l\right)*log{\left(1-p\right)}^{-1}+l*log{\left(p\right)}^{-1}, \end{array}$$

where *p* is the prediction probability of a candidate region with a face.

*Landmark localization*. We consider the category of candidate regions and the visibility factor of landmark points when computing the loss function of landmark localization. There is no loss corresponding to invisible landmark points or negative candidate regions. We compute the loss of landmark location by

(2) $$\begin{array}{l} loss_{L}=\frac{l}{2N_{l}}\overset{N_{l}}{\underset{i=1}{\sum }}v_{i}\left({\left({\hat{a}}_{i}-a_{i}\right)}^{2}+{\left({\hat{b}}_{i}-b_{i}\right)}^{2}\right), \end{array}$$

where $\left({â}_{i},{\hat{b}}_{i}\right)$ is the *i*^*th*^ predicted landmark location. If the *i*^*th*^ landmark is visible in the positive candidate region, the visibility factor *v*_*i*_ is 1; otherwise, it is 0. *N*_*l*_ is the total number of landmark points in a candidate region.

*Landmark visibility*. This task is learned with positive regions to estimate the presence of the predicted landmark. The loss function is shown in (3)

(3) $$\begin{array}{l} loss_{V}=\frac{l}{N_{l}}\underset{i=1}{\sum }N_{l}{\left({\hat{v}}_{i}-v_{i}\right)}^{2}, \end{array}$$

where ${\hat{v}}_{i}$ is the predicted visibility of the *i*^*th*^ landmark.

*Pose estimation*. The head pose annotation contains roll, pitch, and yaw expressed as (*p*_1_, *p*_2_, *p*_3_) in ground truth. We compute the loss of pose estimation for a positive candidate region by

(4) $$\begin{array}{l} loss_{P}=\frac{l}{3}\left({\left({\hat{p}}_{1}-p_{1}\right)}^{2}+{\left({\hat{p}}_{2}-p_{2}\right)}^{2}+{\left({\hat{p}}_{3}-p_{3}\right)}^{2}\right), \end{array}$$

where $\left({\hat{p}}_{1},{\hat{p}}_{2},{\hat{p}}_{3}\right)$ are the pose estimations.

*Gender classification*. Predicting gender is a two-class problem similar to face detection. Computing the loss of the gender prediction for a positive candidate region is defined as

(5) $$\begin{array}{l} loss_{G}=l\left(1-g\right)*log{\left(1-p_{g}\right)}^{-1}+lg*log{\left(p_{g}\right)}^{-1}, \end{array}$$

where *g* = 0 if the gender is male, or else *g* = 1. *p*_*g*_ is the predicted probability of male.

**Trading off loss**. The simple approach to combining losses among learning tasks is to directly perform a linear weighted sum of the losses for each individual task, as shown in (6)

(6) $$\begin{array}{l} loss_{all}=\overset{5}{\underset{i=1}{\sum }}\lambda_{t_{i}}loss_{t_{i}}, \end{array}$$

where *t*_*i*_ is the *i*^*th*^ element from the set of tasks *T* = {*D, L, V, P, G*} and parameter λ_*t*_*i*__ is the weight of each task. However, the naive method of tuning weights manually makes it difficult to balance the performance of individual tasks. We aim to better balance the process of iteratively computing average noisy gradient for each task by using homoscedastic task uncertainty to trade off multiple loss functions. Homoscedastic task uncertainty, which captures the relative confidence between tasks, is a quantity that remains constant for all input data and varies between different tasks, reflecting the uncertainty inherent to the regression or classification task. Homoscedastic uncertainty can be used as a basis for weighting losses in a multi-task learning problem. The positive scalar σ added to the total loss function relates to the uncertainty of the tasks as measured in terms of entropy. The total loss function with the homoscedastic task uncertainty is finally provided by

(7) $$\begin{array}{l} L\left(\lambda_{t_{i}},\sigma_{1},\sigma_{2},\ldots ,\sigma_{i}\right)=\overset{5}{\underset{i=1}{\sum }}\frac{1}{2\sigma_{i}^{2}}L_{t_{i}}\left(\lambda_{t_{i}}\right)+log\sigma_{i}^{2} \end{array}$$

**Privacy accounting**. For our DPLH model, we attach importance to computing the overall privacy cost of training. When iteratively computing the average noisy gradient for each task, the composability of differential privacy allows the privacy accountant to accumulate the privacy cost corresponding to all of the gradients. To make the testing process more transparent and to ensure our model provides a (ε, δ)-differential privacy guarantee, we encapsulate the key differential privacy mechanism into the privacy accountant and positively tune the hyperparameters to achieve different levels of privacy protection.

#### 3.2.3. Architecture of DPLH

**Figure 2:**
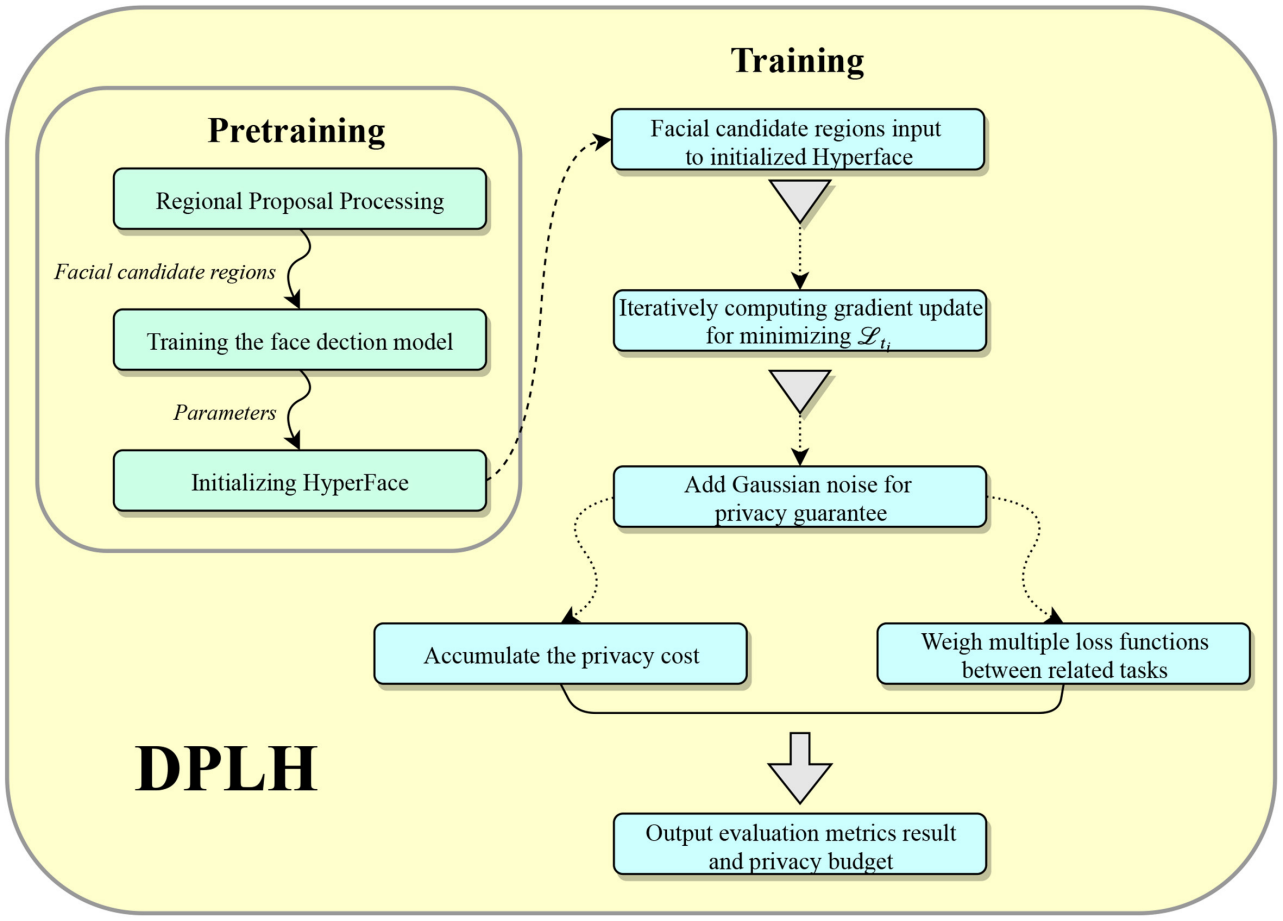
The architecture of DPLH.

In this section, we describe the flow of processing training data in our proposed method, as illustrated in Figure 2.

As shown in Figure 2, the model input is composed of candidate regions with a specific size of (227, 227) generated by the regional candidate selection. Positive candidate regions have full ground truth of face detection, landmark coordinates, landmark visibility factors, pose estimation, and gender information. In contrast, negative candidate regions without faces have the ground truth of face detection, and other ground truths are set to none. These data with ground truth are used to adjust the weights and bias of each layer in the network. In pre-training, we train a single-task model for face detection, and the learned parameters from this network are used to initialize Hyperface. Thereby, we use the candidate regions with adjusted ground truth as input to train the privacy-preserving model. We iteratively compute the gradient update from training data and then apply the Gaussian mechanism for differential privacy to the gradient update, and the privacy cost of iterative calculation is accumulated and accounted. We balance the loss functions of related tasks to ensure better performance for applying the differential privacy mechanism on each task and output a modest small loss. In the end, we will get an output of the evaluation metric results and the privacy budget.

### 3.3. Discussion

The proposed approach, DPLH, aims to preserve private and sensitive information in training datasets. The main idea is to iteratively compute the HyperFace model update from optimizing loss functions and then apply the Gaussian mechanism for differential privacy to the gradient update before incorporating it into the model. In principle, this method can theoretically provide the (ε, δ)-differential privacy guarantee and can prevent private and sensitive data from being maliciously recovered. Furthermore, we use a privacy accountant to estimate the privacy cost of the training process and use different loss functions and evaluation metrics for the training tasks of face detection, landmark localization, landmark visibility, pose estimation, and gender classification. In the end, the losses of each task have reasonable, small values, and the evaluation metrics of each loss function will reflect good performance.

## 4. Experiment

In this section, we evaluate our approach on the AFLW dataset (Martin Koestinger and Bischof, 2011) and report the results of each task for different noise levels. Section 4.1 introduces the details of the experimental setup and the training dataset. Sections 4.2 and 4.3 show the results and analysis.

### 4.1. Dataset and Experimental Setup

We train our model by using the AFLW dataset, which contains more than 25,000 faces in almost 22,000 real-world images with full poses, gender variations, and some more private information. It provides 21 landmark point coordinates per face, along with the face bounding-box, face pose (yaw, pitch, and roll), and gender information. These data cannot be directly used as inputs to the model. We need to prepare the input of the model for evaluating face detection, landmark localization, landmark visibility, pose estimation, and gender classification.

The input does not come from the original dataset, AFLW, but rather comprises candidate regions generated by the regional candidate selection method. The proposed method introduced in section 3 is used for cropping essential regions from images and adjusting privacy-related facial features. For each image from the AFLW dataset, we use the Selective Search (Van de Sande et al., 2011) algorithm to generate candidate regions for faces and then filter out positive samples and negative samples by computing the Intersection over Union (IOU) overlap. The equation of IOU is

(8) $$\begin{array}{l} IOU=\frac{A_{overlap}}{A_{union}} \end{array}$$

where *A*_*overlap*_ is the area of overlap between the selected candidate region and the ground truth bounding-box, and *A*_*union*_ is the area of union encompassed by both of them. Positive candidate regions are selected from regions that have an IOU overlap of more than 0.5 with the ground truth bounding box. The candidate regions with an IOU overlap of <0.35 are considered as negative candidate regions, and other candidate regions are neglected. Subsequently, we scale these selected candidate regions uniformly to 227 * 227 pixels to match the input size of our model. Note that the faces in the images have full pose variations, resulting in some of the landmark points being invisible. We use a visibility factor to annotate visible landmarks provided by the AFLW dataset (Martin Koestinger and Bischof, 2011). However, the given ground truth fiducial coordinates and corresponding visibility factors are relative to the original images. Training the model directly by using the raw information can have a negative impact on the quality of the model. Hence, the landmark points are shifted and scaled to the selected candidate regions using (9)

(9) $$\begin{array}{l} \left(a_{i},b_{i}\right)=\left(\frac{c_{i}-c}{w}*w^{\prime },\frac{d_{i}-d}{h}*h^{\prime }\right) \end{array}$$

where ${\left(c_{i},d_{i}\right)}^{\prime }$s are the given ground truth fiducial coordinates, and ${\left(a_{i},b_{i}\right)}^{\prime }$s are the ground truth fiducial coordinates of adjusted candidate regions. These regions can be characterized by {*c, d, w, h*}, where (*c, d*) are the upper left coordinates of a region and *w, h* are the width and height of the region, respectively. In the end, some of the visible landmark are modified to be invisible, because positive candidate regions may not contain all ${\left(a_{i},b_{i}\right)}^{\prime }$s. The landmark points of negative candidate regions are set to invisible by default.

In our experiments, we obtain more than 40,000 candidate regions. We take 70% of them to train models and the rest for evaluating model performance. Moreover, we set some hyperparameters to fixed values for the next experiments. The sampling probability is given by *q* = *L*/*N*_*c*_, where *N*_*c*_ is the total number of inputs and *L* is the number of samples involved in a batch. We fix the clipping threshold *S* = 0.5, the number of epochs *E* = 500, batch size *L* = 32, input size *N*_*c*_ = 40, 000, and the learning rate η = 0.00015.

### 4.2. Results of Model Training

In this experiment, we compare the results of our model DPLH training and HyperFace training. In order to better evaluate the performance of each task, we choose the accuracy metrics for face detection and gender classification and the mean square error metrics for landmark localization, landmark visibility, and pose estimation. We allocate the privacy budget (ε, δ) as (5, 10^−5^) to DPLH to provide a privacy guarantee.

**Figure 3:**
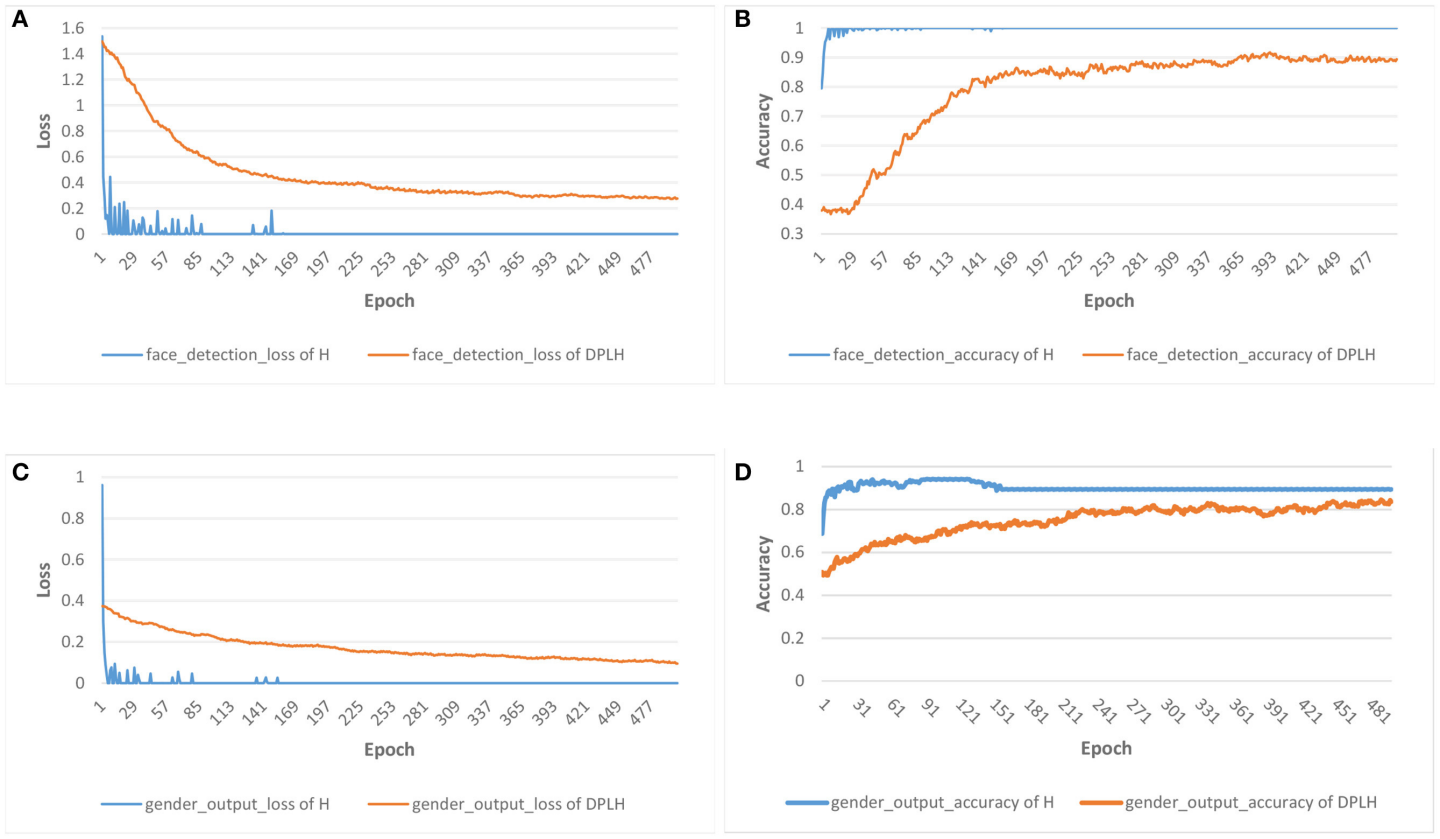
Results for the loss and accuracy of face detection and gender classification on HyperFace (H) vs. DPLH. **(A)** Loss of face detection. **(B)** Accuracy of face detection. **(C)** Loss of gender classification. **(D)** Accuracy of gender classification.

**Figure 4:**
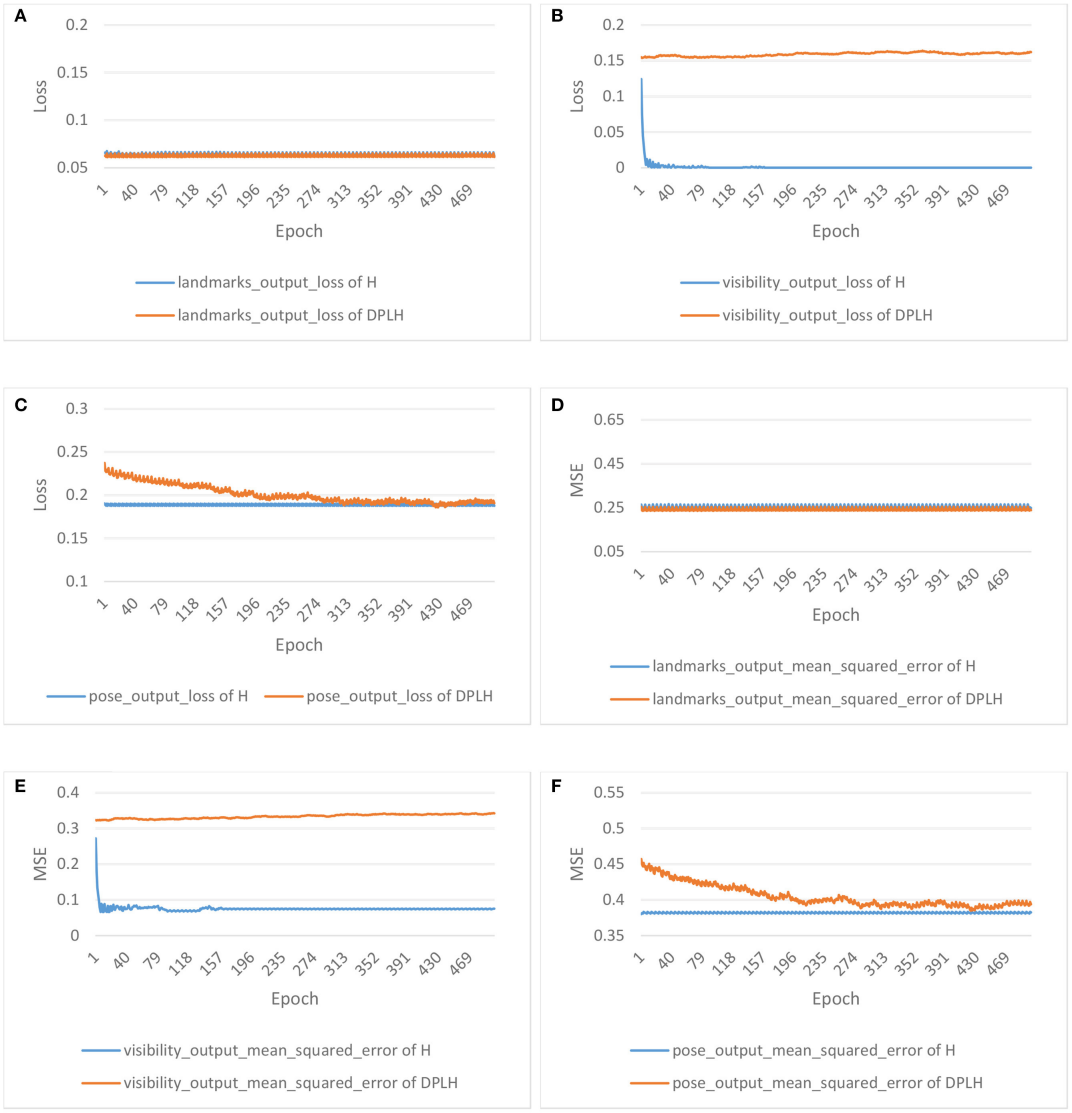
Results for the loss and mean square error (MSE) of landmark localization, landmark visibility, and pose estimation on HyperFace(H) vs. DPLH. **(A)** Loss of landmark localization. **(B)** Loss of landmark visibility. **(C)** Loss of pose estimation. **(D)** MSE of landmark localization. **(E)** MSE of landmark visibility. **(F)** MSE of pose estimation.

Figure 3 shows the results for the loss and accuracy of face detection and gender classification on the two models. A declining trend of losses is depicted in Figure 3A for face detection and Figure 3C for gender classification. As the epochs increase in number, the losses of HyperFace on these tasks decline faster, and the losses of DPLH decrease gently. After convergence, the two models consume 500 epochs to reduce the loss to desirably small values. Moreover, the losses from training HyperFace converge to a smaller level than the differentially private losses. Additionally, Figures 3B,D illustrate the growth trend of accuracy for face detection and gender classification, respectively. The accuracy from training HyperFace consuming the same number of epochs rises fastest, and, in addition, the metrics evaluating the two models both converge to high levels. Figure 4 shows the results for the loss and mean square error (MSE) of landmark localization, landmark visibility, and pose estimation on the two models. Similar to Figure 3, the losses of the three tasks on training the two models converge to desirably small levels. The MSE curves decline to small values, converging to a nearby level, respectively on their tasks.

These figures indicate that the final results for loss, accuracy, and mean square error converge to a desirable level. From the perspective of three metrics, the two models can almost achieve approximate results on respective tasks, which demonstrates that our approach decreases model performance and utility very little compared with HyperFace. Our approach achieves 90 and 86% accuracy on face detection and gender classification, respectively, compared with 99 and 90% accuracy on HyperFace. For landmark localization, landmark visibility, and pose estimation, our approach achieves 0.255, 0.25, and 0.27 mean square error, respectively, compared with 0.245, 0.2, and 0.24 on HyperFace. The final results indicate that our approach can provide a differential privacy guarantee with desirable performance of the system. We conduct a *t*-test on the performance of multiple tasks with different epochs. For *p*-value ≤ 0.05, the performance of the DPLH method approximates to that of Hyperface without privacy preservation. As shown in Table 2, the extremely small *p*-value indicates that the DPLH method provides a differential privacy guarantee and achieves performance that is similar to that of the Hyperface method.

**Table 2:** *T*-test results on the performance of multiple tasks at different epochs.

**Epochs**	**Task**	**50**	**100**	**150**	**200**	**250**	**300**	**350**	**400**	**450**	**500**
*P*-value (%)	*loss*_*D*_	0.068	0.054	0.039	0.033	0.032	0.031	0.031	0.031	0.030	0.030
	*loss*_*L*_	0.185	0.123	0.105	0.089	0.075	0.073	0.073	0.072	0.072	0.072
	*loss*_*V*_	0.314	0.285	0.212	0.183	0.179	0.178	0.178	0.178	0.178	0.178
	*loss*_*P*_	0.351	0.317	0.297	0.283	0.279	0.279	0.279	0.279	0.279	0.279
	*loss*_*G*_	0.063	0.057	0.049	0.039	0.038	0.038	0.038	0.038	0.038	0.038
	*Acc*_*D*_	0.093	0.078	0.061	0.057	0.056	0.054	0.054	0.054	0.054	0.054
	*Acc*_*G*_	0.045	0.031	0.027	0.025	0.024	0.024	0.024	0.024	0.024	0.024
	*MSE*_*L*_	0.194	0.172	0.151	0.143	0.139	0.138	0.138	0.138	0.138	0.138
	*MSE*_*V*_	0.112	0.089	0.073	0.067	0.065	0.065	0.065	0.065	0.065	0.065
	*MSE*_*P*_	0.185	0.169	0.154	0.147	0.145	0.145	0.145	0.145	0.145	0.145

### 4.3. Results for Training With Different Noise Levels

In this experiment, we consider the effect of different noise levels on the performance of DPLH. We compare three noise levels for the training characteristics of HyperFace integrated with differential privacy. We set a privacy budget ε = 0.7 to train the DPLH with a number of epochs *E* = 500, which represents high noise level training. Besides, we consume a fixed ε = 5 privacy budget per epoch to train HyperFace with a modest noise level. Moreover, low noise level training is performed on HyperFace with a privacy budget ε = 10 per epoch. In addition, that we fix δ = 10^−5^ per figure.

**Figure 5:**
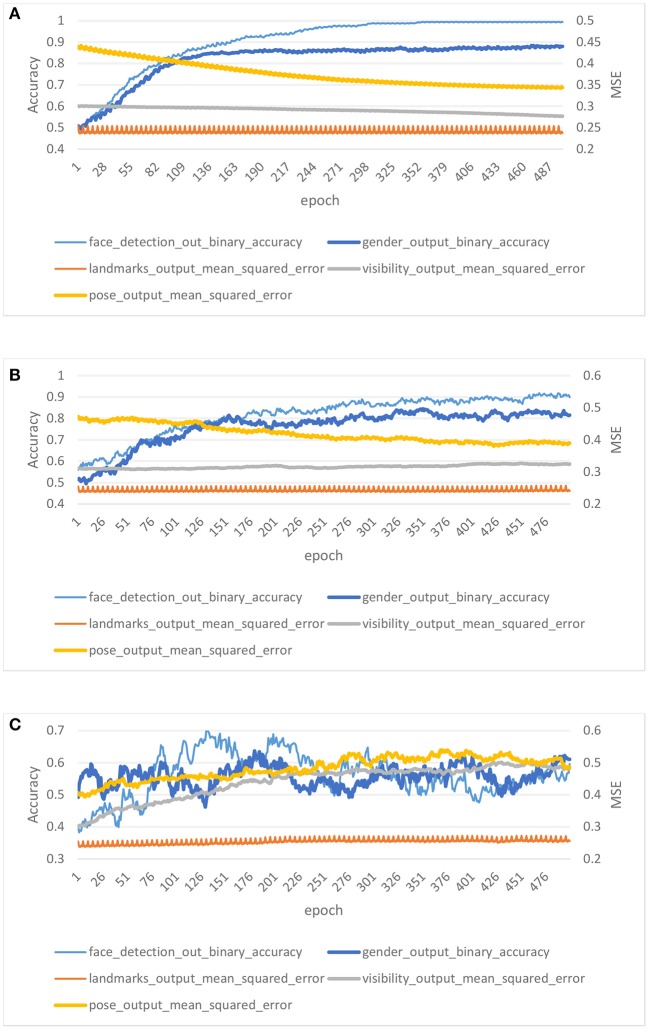
Results for low noise level training, modest noise level training, and high noise level training with privacy budget (10, 10^−5^), (5, 10^−5^), (0.7, 10^−5^). **(A)** Low noise level. **(B)** Modest noise level. **(C)** High noise level.

Figure 5 shows the results on different privacy budgets (ε, δ). In each plot, we show the evaluation of accuracy for two tasks (face detection and gender classification) and the mean square error for three tasks (landmark localization, landmark visibility, and pose estimation). Figures 5A,B illustrate low noise level training and modest noise level training, respectively. The accuracy of the two noise levels rises gently, and the accuracy of low noise level training is higher than that of modest noise level training after convergence. On the evaluation of MSE, the two noise level trainings converge to a desirable level. In contrast, Figure 5C illustrates high noise level training performance on DPLH. The accuracy of high noise level training converges to lower values, and the MSE shows a unstable decline trend. We achieve desirable performance for (10, 10^−5^), (5, 10^−5^) differential privacy, respectively, since the accuracy converges to a high level and the MSE converges to a low level. However, (0.7, 10^−5^)-differential privacy training brings too much noise to the model, resulting in unstable performance. The final results indicate that acceptable noise level training on HyperFace can provide a differential privacy guarantee and stable performance, while an excessive noise level may destroy the performance and utility of the model, making privacy preservation irrelevant.

## 5. Conclusion

In this paper, we propose a novel method called differentially private learning on HyperFace that provides a differential privacy guarantee and desirable performance for simultaneously learning face detection, landmark localization, pose estimation, and gender classification. We demonstrate the utility and effectiveness of our model for training all four tasks on the datasets. In the future, we will carry out further studies on selecting the most appropriate noise level automatically to provide a differential privacy guarantee and excellent performance.

## Data Availability Statement

The datasets analyzed in this manuscript are not publicly available. Requests to access the datasets should be directed to huhsiungwei@gmail.com.

## Author Contributions

YX and XH conceptualized the problem and the technical framework. MG and CZ developed the algorithms, supervised the experiments, and exported the data. YX, XH, and BY implemented the privacy-preserving multi-task learning architecture simulation. BY managed the project. All of the authors wrote the manuscript, discussed the experimental results, and commented on the manuscript.

### Conflict of Interest

The authors declare that the research was conducted in the absence of any commercial or financial relationships that could be construed as a potential conflict of interest.

## References

[B1] Abadi M. Chu A. Goodfellow I. McMahan H. B. Mironov I. Talwar K. (2016). “Deep learning with differential privacy,” in Proceedings of the 2016 ACM SIGSAC Conference on Computer and Communications Security (Vienna: ACM), 308–318.

[B2] Ahn B. Choi D.-G. Park J. Kweon I. S. (2018). Real-time head pose estimation using multi-task deep neural network. Robot. Auton. Syst. 103, 1–12. 10.1016/j.robot.2018.01.005

[B3] Bun M. Dwork C. Rothblum G. N. Steinke T. (2018). “Composable and versatile privacy via truncated CDP,” in Proceedings of the 50th Annual ACM SIGACT Symposium on Theory of Computing (Los Angeles, CA: ACM), 74–86.

[B4] Chaudhuri K. Monteleoni C. Sarwate A. D. (2011). Differentially private empirical risk minimization. J. Mach. Learn. Res. 12, 1069–1109. 21892342 PMC3164588

[B5] Chaudhuri K. Sarwate A. D. Sinha K. (2013). A near-optimal algorithm for differentially-private principal components. J. Mach. Learn. Res. 14, 2905–2943.

[B6] Chen J.-C. Lin W.-A. Zheng J. Chellappa R. (2018). “A real-time multi-task single shot face detector,” in 2018 25th IEEE International Conference on Image Processing (ICIP) (Athens: IEEE), 176–180.

[B7] Corinzia L. Buhmann J. M. (2019). Variational federated multi-task learning. arXiv 1906.06268.

[B8] Doersch C. Zisserman A. (2017). “Multi-task self-supervised visual learning,” in Proceedings of the IEEE International Conference on Computer Vision (Venice), 2051–2060.

[B9] Dwork C. (2011a). Differential privacy. Encycl. Cryptogr. Secur. 338–340. 10.1007/978-1-4419-5906-5_752

[B10] Dwork C. (2011b). A firm foundation for private data analysis. Commun. ACM 54, 86–95. 10.1145/1866739.1866758

[B11] Dwork C. Kenthapadi K. McSherry F. Mironov I. Naor M. (2006a). “Our data, ourselves: privacy via distributed noise generation,” in Annual International Conference on the Theory and Applications of Cryptographic Techniques (St. Petersburg: Springer), 486–503.

[B12] Dwork C. McSherry F. Nissim K. Smith A. (2006b). “Calibrating noise to sensitivity in private data analysis,” in Theory of Cryptography Conference (New York, NY: Springer), 265–284.

[B13] Dwork C. Roth A. (2014). The algorithmic foundations of differential privacy. Found. Trends Theor. Comput. Sci. 9, 211–407. 10.1561/0400000042

[B14] Eigen D. Fergus R. (2015). “Predicting depth, surface normals and semantic labels with a common multi-scale convolutional architecture,” in Proceedings of the IEEE International Conference on Computer Vision (Santiago), 2650–2658.

[B15] Erlingsson Ú. Feldman V. Mironov I. Raghunathan A. Talwar K. Thakurta A. (2019). “Amplification by shuffling: from local to central differential privacy via anonymity,” in Proceedings of the Thirtieth Annual ACM-SIAM Symposium on Discrete Algorithms (San Diego, CA: SIAM), 2468–2479.

[B16] Gupta S. K. Rana S. Venkatesh S. (2016). “Differentially private multi-task learning,” in Pacific-Asia Workshop on Intelligence and Security Informatics (Springer), 101–113.

[B17] Han H. Jain A. K. Wang F. Shan S. Chen X. (2017). Heterogeneous face attribute estimation: a deep multi-task learning approach. IEEE Trans. Pattern Anal. Mach. Intell. 40, 2597–2609. 10.1109/TPAMI.2017.2738004 28809673

[B18] Hessel M. Soyer H. Espeholt L. Czarnecki W. Schmitt S. van Hasselt H. (2019). “Multi-task deep reinforcement learning with Popart,” in Proceedings of the AAAI Conference on Artificial Intelligence (Hawaii), Vol. 33, 3796–3803.

[B19] Huang J.-T. Li J. Yu D. Deng L. Gong Y. (2013). “Cross-language knowledge transfer using multilingual deep neural network with shared hidden layers,” in 2013 IEEE International Conference on Acoustics, Speech and Signal Processing (Vancouver, BC: IEEE), 7304–7308.

[B20] Kim S. Hori T. Watanabe S. (2017). “Joint ctc-attention based end-to-end speech recognition using multi-task learning,” in 2017 IEEE International Conference on Acoustics, Speech and Signal Processing (New Orleans, LA: IEEE), 4835–4839.

[B21] Kokkinos I. (2017). “Ubernet: training a universal convolutional neural network for low-, mid-, and high-level vision using diverse datasets and limited memory,” in Proceedings of the IEEE Conference on Computer Vision and Pattern Recognition (Hawaii), 6129–6138.

[B22] Li N. Li T. Venkatasubramanian S. (2007). “t-Closeness: privacy beyond k-anonymity and l-diversity,” in Proceedings of 23rd International Conference on Data Engineering (Istanbul: IEEE), 106–115.

[B23] Liu K. Uplavikar N. Jiang W. Fu Y. (2018). “Privacy-preserving multi-task learning,” in IEEE International Conference on Data Mining (Singapore), 1128–1133.

[B24] Liu P. Qiu X. Huang X. (2017). Adversarial multi-task learning for text classification. arXiv 1704.05742. 10.18653/v1/P17-1001

[B25] Liu S. Johns E. Davison A. J. (2019). “End-to-end multi-task learning with attention,” in Proceedings of the IEEE Conference on Computer Vision and Pattern Recognition (Long Beach, CA), 1871–1880.

[B26] Machanavajjhala A. Gehrke J. Kifer D. Venkitasubramaniam M. (2006). “l-Diversity: Privacy beyond k-anonymity,” in Proceedings of 22nd International Conference on Data Engineering (ICDE'06) (Atlanta, GA: IEEE), 24–24.

[B27] Martin Koestinger Paul Wohlhart P. M. R. Bischof H. (2011). “Annotated facial landmarks in the wild: a large-scale, real-world database for facial landmark localization,” in Proceedings of 1st IEEE International Workshop on Benchmarking Facial Image Analysis Technologies (Barcelona).

[B28] McMahan H. B. Ramage D. Talwar K. Zhang L. (2017). Learning differentially private recurrent language models. arXiv 1710.06963.

[B29] McSherry F. Talwar K. (2007). Mechanism design via differential privacy. FOCS 7, 94–103. 10.1109/FOCS.2007.66

[B30] Papernot N. Abadi M. Erlingsson U. Goodfellow I. Talwar K. (2016). Semi-supervised knowledge transfer for deep learning from private training data. arXiv 1610.05755.

[B31] Phan N. Wu X. Hu H. Dou D. (2017). “Adaptive laplace mechanism: differential privacy preservation in deep learning,” in 2017 IEEE International Conference on Data Mining (ICDM) (New Orleans, LA: IEEE), 385–394.

[B32] Ranjan R. Patel V. M. Chellappa R. (2017). Hyperface: a deep multi-task learning framework for face detection, landmark localization, pose estimation, and gender recognition. IEEE Trans. Pattern Anal. Mach. Intell. 41, 121–135. 10.1109/TPAMI.2017.2781233 29990235

[B33] Sattler F. Müller K.-R. Samek W. (2019). Clustered federated learning: model-agnostic distributed multi-task optimization under privacy constraints. arXiv 1910.01991. 10.1109/TNNLS.2020.3015958 32833654

[B34] Sermanet P. Eigen D. Zhang X. Mathieu M. Fergus R. LeCun Y. (2013). Overfeat: integrated recognition, localization and detection using convolutional networks. arXiv 1312.6229.

[B35] Smith V. Chiang C.-K. Sanjabi M. Talwalkar A. S. (2017). “Federated multi-task learning,” in Advances in Neural Information Processing Systems (Long Beach, CA), 4424–4434.

[B36] Subramanian S. Trischler A. Bengio Y. Pal C. J. (2018). Learning general purpose distributed sentence representations via large scale multi-task learning. arXiv 1804.00079.

[B37] Sweeney L. (2002). k-Anonymity: a model for protecting privacy. Int. J. Uncertainty Fuzziness Knowl. Based Syst. 10, 557–570. 10.1142/S0218488502001648

[B38] Van de Sande K. E. Uijlings J. R. Gevers T. Smeulders A. W. (2011). “Segmentation as selective search for object recognition,” in Proceedings of 11th IEEE International Conference on Computer Vision, Vol. 1 (Barcelona), 7.

[B39] Wang K. Chen R. Fung B. Yu P. (2010). Privacy-preserving data publishing: a survey on recent developments. ACM Comput. Surveys 42:14 10.1145/1749603.1749605

[B40] Wang Y.-X. Balle B. Kasiviswanathan S. (2018). Subsampled rényi differential privacy and analytical moments accountant. arXiv 1808.00087.

[B41] Wu X. Li F. Kumar A. Chaudhuri K. Jha S. Naughton J. (2017). “Bolt-on differential privacy for scalable stochastic gradient descent-based analytics,” in Proceedings of the 2017 ACM International Conference on Management of Data (Chicago, IL: ACM), 1307–1322.

[B42] Xie L. Baytas I. M. Lin K. Zhou J. (2017). “Privacy-preserving distributed multi-task learning with asynchronous updates,” in Proceedings of the 23rd ACM SIGKDD International Conference on Knowledge Discovery and Data Mining (Halifax, NS: ACM), 1195–1204.

[B43] Yin X. Liu X. (2017). Multi-task convolutional neural network for pose-invariant face recognition. IEEE Trans. Image Process. 27, 964–975. 10.1109/TIP.2017.2765830 29757739

[B44] Zhao Y. Tang F. Dong W. Huang F. Zhang X. (2019). Joint face alignment and segmentation via deep multi-task learning. Multimed. Tools Appl. 78, 13131–13148. 10.1007/s11042-018-5609-1

